# Radiomics Signatures Based on Computed Tomography for Noninvasive Prediction of *CXCL10* Expression and Prognosis in Ovarian Cancer

**DOI:** 10.1002/cnr2.70030

**Published:** 2024-10-23

**Authors:** Xiaohua Wang, Yuanyuan Xing, Xuan Zhou, Chunhui Wang, Shuyu Han, Sufen Zhao

**Affiliations:** ^1^ Department of Gynecology and Obstetrics, Department of Gynecology The Second Hospital of HeBei Medical University, Affiliated Hospital of Chengde Medical University Shijiazhuang China; ^2^ Department of Nuclear Medicine Affiliated Hospital of Chengde Medical University Chengde China; ^3^ Department of Gynecology Affiliated Hospital of Chengde Medical University Chengde China; ^4^ Department of Gynecology and Obstetrics The Second Hospital of HeBei Medical University Shijiazhuang China

**Keywords:** CXCL10, ovarian cancer, predictive model, prognosis, radiomics

## Abstract

**Background:**

Ovarian cancer (OC) is an aggressive gynecological tumor usually diagnosed with malignant ascites and even observed widespread metastasis or distant spread.

**Aims:**

We aimed to develop and identify radiomics models according to computed tomography (CT) for preoperative prediction of *CXCL10* expression and prognosis in patients with OC.

**Methods:**

Genomic data with CT images and corresponding clinicopathological parameters were extracted from The Cancer Imaging Archive (TCIA) and The Cancer Genome Atlas (TCGA). To analyze the prognosis, we carried out the univariate Cox regression analysis (UCRA), multivariate Cox regression analysis (MCRA), and Kaplan–Meier (KM) analysis. For the data reduction, logistic regression, operator regression, least absolute shrinkage selection, radiomic feature construction, and feature selection were utilized. The predictive performance of the radiomic signatures was assessed using the analyses of the receiver operating characteristic (ROC) curve, decision curve (DCA), and precision‐recall (PR) curve. To evaluate the correlation between the radiomic score (Rad‐score) and *CXCL10* expression, the Wilcoxon rank‐sum test was applied.

**Results:**

Three radiomics models effectively predicted *CXCL10* expression levels (AUC = 0.791, 0.748, and 0.718 for the set of training; AUC = 0.761, 0.746, and 0.701 for the set of validation). A higher Rad‐score significantly correlated with upregulated *CXCL10* expression.

**Conclusion:**

*CXCL10* expression can be predicted noninvasively and preoperatively via radiomic signatures based on contrast‐enhanced CT images.

## 1 Introduction

Among all gynecological cancers, the 5‐year survival rate of patients with ovarian cancer (OC) no more than 46% [1]. According to estimates, there were more than 12 810 deaths and 19 880 new cases in the United States in 2022 [2]. A large proportion of patients with OC are diagnosed with advanced disease, often involving the pelvic and abdominal cavity, with malignant ascites, and even observed transluminal metastasis or distant spread. The established protocol for initial OC treatment involves platinum‐based chemotherapy along with cytoreductive surgery, optionally complemented by administrating bevacizumab (the angiogenesis inhibitor) [3]. This is followed by a maintenance regimen incorporating poly (ADP‐ribose) polymerase (PARP) inhibitors (PARPi) [4, 5]. Regrettably, a substantial proportion of patients with OC, particularly those with stage‐III/IV high‐grade serous OC (HGSC), experiences a recurrence of 70% within 5 years [6, 7]. Owing to the high recurrence rates and chemotherapy resistance, traditional therapeutic options exhibit inadequate efficacy [8]. The diagnosis of OC includes MRI examination, pelvic computed tomography (CT), human epididymis protein 4 (HE4), or serum carbohydrate antigen 125 (CA125) [9, 10]. Histopathological examinations can only be performed postoperatively as a diagnostic criterion. Therefore, developing effective early diagnostic strategies and therapeutics for patients with OC is essential.

Chemokines C‐X‐C motif chemokine ligand 10 (*CXCL10*) is a CXC chemokine subfamily member, which binds to the CXCR3 (C‐X‐C motif chemokine receptor 3) to perform its biological functions [11]. *CXCL10* plays multiple roles in regulation of the immune response and the tumor development by participating the interactive processes of immune and cancer cells [12, 13]. *CXCL10* plays a crucial role in immune cell migration, differentiation, and activation [14]. Several studies have demonstrated that increased *CXCL10* expression correlates with improved prognosis in various human cancers, including high‐grade serous ovarian carcinoma, colorectal cancer (CRC), hepatocellular carcinoma, and breast carcinoma [15, 16, 17, 18]. Evidence shows a significant connection of the *CXCL10* expression to the development of tumors, signature of tumor microenvironment, infiltration of immune cells, alterations of genetics, and the prognosis of the patient [19, 20, 21]. However, there is currently no noninvasive tool to assess CXCL10 mRNA expression levels, which can only be detected via surgical resection or invasive biopsy in OC.

Radiomics, a technique for analyzing complex medical images using sophisticated image analysis tools, can improve the accuracy of prediction, prognosis, and diagnosis and plays an increasingly crucial role in cancer research [22, 23, 24, 25]. Radiomics can eliminate the influence of subjective observer factors on the diagnostic results, ensure the stability of diagnostic efficiency, and support clinical decision‐making [26]. Le et al. [27] developed the risk scores based on 10 radiomics signatures models in CT images demonstrated great potential in predicting the overall survival of patients with nonsmall cell lung cancer. CT is commonly applied in the diagnosis, screening, prediction, treatment planning, and treatment response evaluation for OC management [28]. Hobbs et al. [29] observed a significant correlation of the radiographic findings, including contrast between nonenhanced and enhanced regions in CT scans, with the major differences in tumor protein expression patterns. Despite major advances in radiogenomics have obtained, the connections of the previously described *CXCL10* molecular subtypes to the radiomic features of CT are not examined. In this study, for predicting the prognosis, preoperative diagnosis, and *CXCL10* expression in patients with OC, we developed and validated a noninvasive radiomic signature based on enhanced CT images.

## 2 Materials and Methods

### 2.1 Acquisition of Data

**FIGURE 1:**
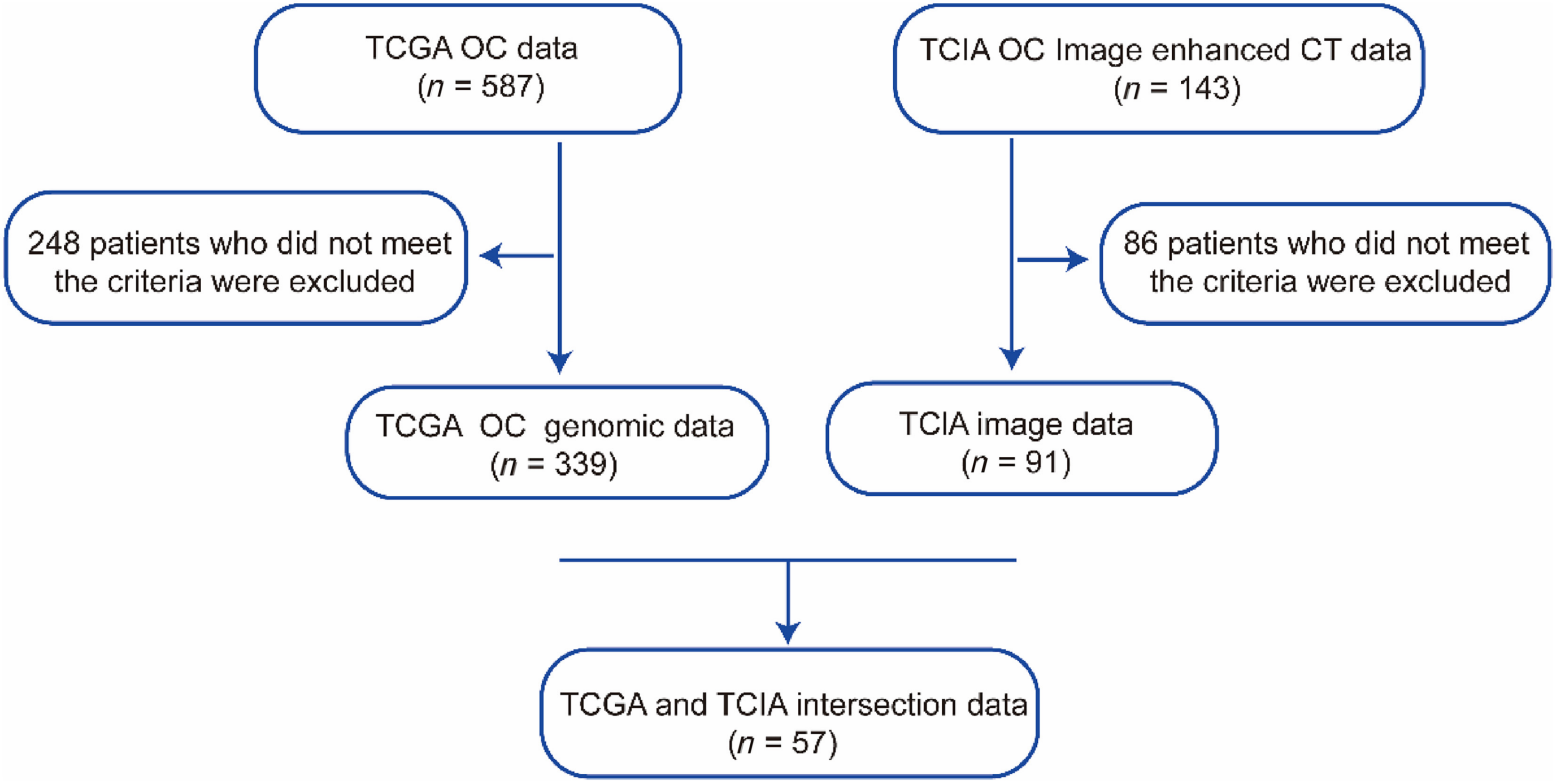
Data collection workflow.

Using the Cancer Genome Atlas (TCGA) (https://portal.gdc.cancer.gov/) and The Cancer Imaging Archive (TCIA) (https://www.cancerimagingarchive.net/) databases [30, 31], 587 OC patients' data about transcriptome sequencing and 143 patients' data about medical images, such as the follow‐up and clinical data, were obtained. The inclusion criteria for patients with OC were as follows: (1) serous cystadenocarcinoma at the first diagnosis and treatment; (2) available RNA‐seq gene expression information; and (3) corresponding clinical data available in the TCIA database. The exclusion criteria for the OC patients were as follows: (1) absence of survival status or duration of follow‐up; (2) cases with overall survival (OS) < 30 days; (3) unknown International Federation of Gynecology and Obstetrics (FIGO) stage and histopathological grade; and (4) CT image quality not up to standard. Figure 1 shows the flowchart of this process.

As covariates, venous invasion (no vs. yes vs. unknown), tumor residual disease (no macroscopic disease vs. 1–10 mm vs. 10 mm vs. unknown), chemotherapy (no vs. yes), histologic grade (G1/G2 vs. G3/G4/GX), FIGO stage (I/II vs. III/IV), lymphatic invasion (no vs. yes vs. unknown), and age (≤ 59 vs. ≥ 60), were included. All the images and data are anonymized and publicly available in TCGA and TCIA; thus, no ethical review and written informed consent are needed for this study.

### 2.2 TCGA Data Analysis

*CXCL10* expression data from the TCGA OC project and normal tissues were extracted from the UCSC XENA and Gene‐Tissue Expression databases. To compare the expression between samples, the Toil process was conducted to transform all of the obtained data into the format of transcript per million (TPM), and subsequently, we converted the values of TPM into log2 (TPM + 1) scales [32]. According to the *CXCL10* expression, we divided all the enrolled cases into two different groups (low and high *CXCL10* groups).

To evaluate the OS among various groups, Kaplan–Meier (KM) curves were generated. The package “forest plot” was employed to complete the univariate Cox regression analysis (UCRA) and multivariate Cox regression analysis (MCRA). The UCRA was used to analyze the effect of the principal variable, *CXCL10*, on the prognosis of patients in different subgroups. The OC gene expression matrix was uploaded to the ImmunecellAI database [33], and immune cell infiltration was calculated in each sample. The correlation between the immune cell infiltration, immune checkpoints, and *CXCL10* expression was analyzed using Spearman's correlation test. Correlation analysis: bubble plots show results for *p* < 0.05.

Using the Hallmark (H.A.v7.5.1. symbols.gmt) and KEGG (c2.cp.kegg.v7.5.1. symbols.gmt) gene sets, we investigated the pathways differentially enriched between the groups of patients with different *CXCL10* expression.

### 2.3 Imaging Data Acquisition and Processing

For the selection of a total of 57 OC samples with intersecting TCGA and TCIA database entries, we applied the package “survminer.” The specimens were categorized into groups based on their CXCL10 expression levels using the cutoff value 5.593, specifically as high or low. A three‐dimensional slicer (version 4.10.2) was employed to import the enhanced CT images, and two radiologists with 10 years of work experience were employed manually to select the volumes of interest under double‐blinded conditions. The radiomic features (*n* = 107) were obtained by PyRadiomics, and then, a normalization of z‐score was conducted [34]. Radiomic features with an intraclass correlation coefficient (ICC) ≥ 0.8 were included for further exploration.

### 2.4 Feature Reduction and Model Establishment

To minimize overfitting, radiomic features were selected using the least absolute shrinkage and selection operator (LASSO) and recursive feature elimination (RFE) methods. We selected features with frequencies > 800 times obtained through 1000 screening LASSO regression features and screened the best feature subset of the two algorithms using a stepwise regression algorithm according to the Akaike information criterion (AIC). We next carried out the logistic regression to build the radiomic model, which transforms the linear regression function into a sigmoid function so that the output value of the model is distributed between zero and one. The radiomics features selected by the RFE_AIC and LASSO_AIC algorithms were fitted using a logistic regression algorithm to establish the RFE_AIC_LR and LASSO_AIC_LR models for predicting CXCL10 expression. The RFE_LASSO_LR model was constructed using the intersection of the features selected by the RFE_AIC and LASSO_AIC algorithms.

**FIGURE 2:**
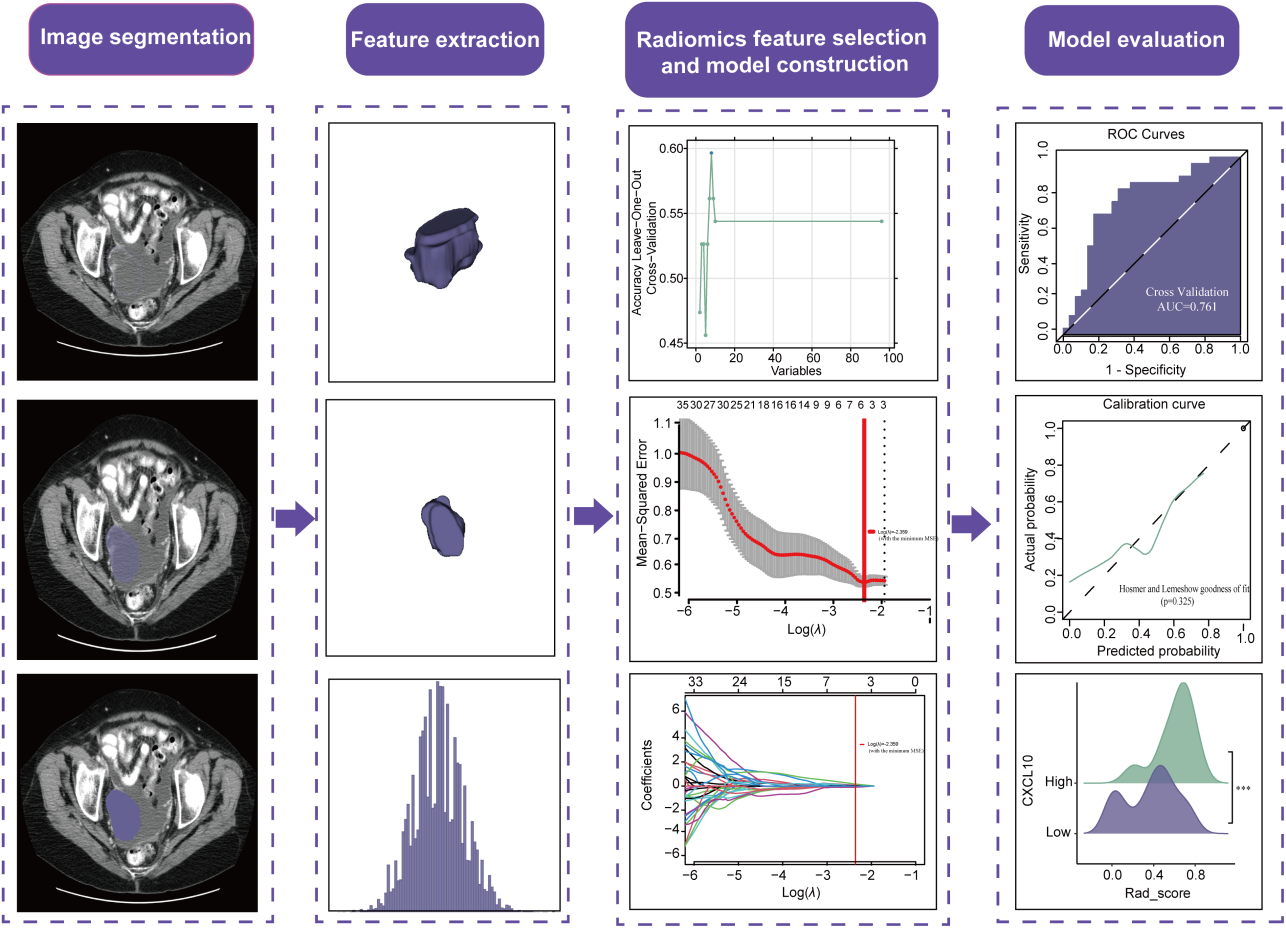
Workflow of the radiomics analysis.

A 10‐fold internal cross‐validation process was used to validate and evaluate radiomics signatures [35]. Using the R packages “pROC” and “modEvA”, we employed the precision‐recall (PR) curves and the receiver operating characteristic (ROC) curves to investigate the performance of the three radiomics models. The diagnostic performance was determined based on these indicators, including the negative predictive value (NPV), sensitivity, specificity, accuracy, positive predictive value (PPV), and the area under the ROC curve (AUC). The radiomics prediction models were calibrated using the Hosmer–Lemeshow test and the calibration curves. The analysis of decision curve (DCA) was employed to evaluate the clinical benefits of the radiomics models using package “rmda.” The radiomics scores (Rad‐scores) between the *CXCL10*‐high and ‐low groups were compared using the Wilcoxon rank‐sum test. Figure 2 shows the workflow of the radiomic analysis.

### 2.5 Statistical Analysis

For analyzing the data, R software (version 4.3.0) was employed. For the analysis of differences between continuous variables, we carried out the Wilcoxon rank‐sum test or Student's *t*‐test. For the analysis of difference between categorical variables, we carried out Fisher's exact test or chi‐square test. To evaluate the survival and generate survival curves, we conducted the package “survival” and Kaplan–Meier analysis. For evaluating the risk factors associated with CXCL10 expression, we carried out the MCRA and UCRA. To evaluate the association of *CXCL10* expression with the infiltration of immune cells and immune checkpoints' expression, as well as the difference of this association, we carried out Spearman's correlation analysis. A significance level of *p* value less than 0.05 was established for statistical analysis.

## 3 Results

### 3.1 Clinical Significance of CXCL10

**FIGURE 3:**
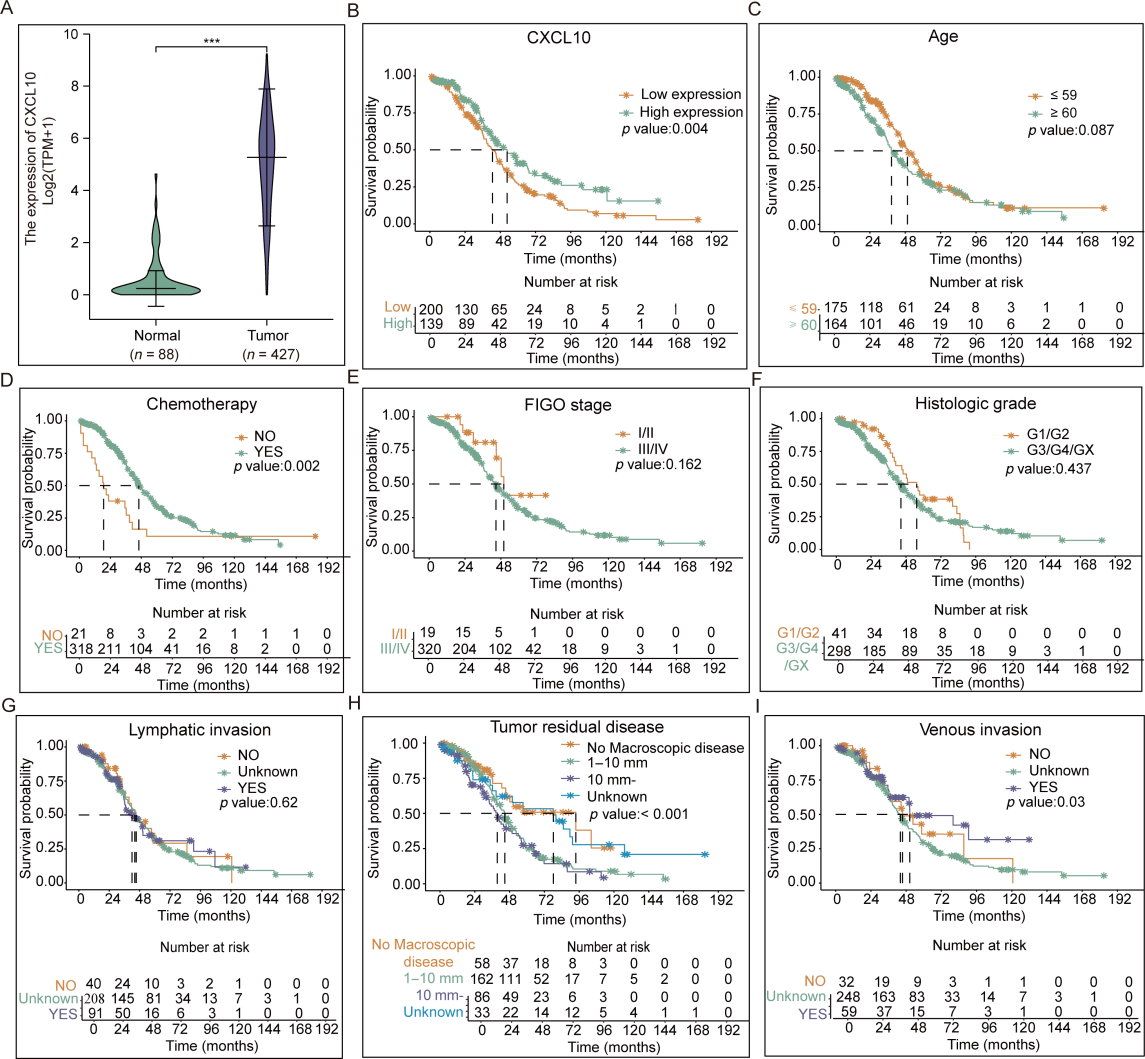
*CXCL10* expression in TCGA and Kaplan–Meier survival curves in subgroups. (A) Compared with 88 normal samples, OC samples (*n* = 427) exhibited significantly elevated CXCL10 transcription. (B, D, H, I) High *CXCL10* expression, chemotherapy, venous invasion, and tumor residual disease were significantly connected to OS in patients with OC. (C, E–G) Histologic grade, lymphatic invasion, FIGO stage, and age were not associated with OS in patients with OC.

As shown in Figure 3A, in comparison to the ovarian samples from normal individuals (*n* = 88), the OC samples (*n* = 427) exhibited significantly elevated CXCL10 transcription (*p* < 0.001). The 339 OC samples that met the inclusion criteria were divided into a *CXCL10*‐high expression group (200) and a *CXCL10*‐low expression group (139). No significant difference in the covariates' distribution (including chemotherapy, tumor residual disease, histological grade, lymphatic invasion, FIGO stage, and age) was observed between the two groups (*p* > 0.05). Clinicopathological data of the patients are presented in Table 1.

**TABLE 1:** Clinicopathological characteristics of TCGA patients with OC.

	Total (*n* = 339)	Low (*n* = 200)	High (*n* = 139)	*p*
Age, *n* (%)				0.7
≤ 59	175 (51.62)	101 (50.5)	74 (53.24)	
≥ 60	164 (48.38)	99 (49.5)	65 (46.76)	
FIGO stage, *n* (%)				0.193
I/II	19 (5.6)	8 (4)	11 (7.91)	
III/IV	320 (94.4)	192 (96)	128 (92.09)	
Venous invasion, *n* (%)				0.037*
No	32 (9.44)	20 (10)	12 (8.63)	
Unknown	248 (73.16)	154 (77)	94 (67.63)	
Yes	59 (17.4)	26 (13)	33 (23.74)	
Lymphatic invasion, *n* (%)				0.054
No	40 (11.8)	25 (12.5)	15 (10.79)	
Unknown	208 (61.36)	131 (65.5)	77 (55.4)	
Yes	91 (26.84)	44 (22)	47 (33.81)	
Histologic grade, *n* (%)				0.434
G1/G2	41 (12.09)	27 (13.5)	14 (10.07)	
G3/G4/GX	298 (87.91)	173 (86.5)	125 (89.93)	
Tumor residual disease, *n* (%)				0.299
No macroscopic disease	58 (17.11)	31 (15.5)	27 (19.42)	
1–10 mm	162 (47.79)	104 (52)	58 (41.73)	
10 mm~	86 (25.37)	48 (24)	38 (27.34)	
Unknown	33 (9.73)	17 (8.5)	16 (11.51)	
Chemotherapy, *n* (%)				0.96
No	21 (6.19)	13 (6.5)	8 (5.76)	
Yes	318 (93.81)	187 (93.5)	131 (94.24)	

* *p* < 0.05.

Meanwhile, in comparison to the patients with low *CXCL10* expression (42.6 months), the patients with high *CXCL10* expression exhibited a relatively long median survival time (52.6 months). High CXCL10 expression and other variables (including chemotherapy, venous invasion, and tumor residual disease) were significantly associated with a protective effect on OS (Figure 3B,D,H,I *p* < 0.05), whereas age, FIGO stage, lymphatic invasion, and histologic grade were not associated with OS in patients with OC (Figure 3C,E–G *p* > 0.05).

**FIGURE 4:**
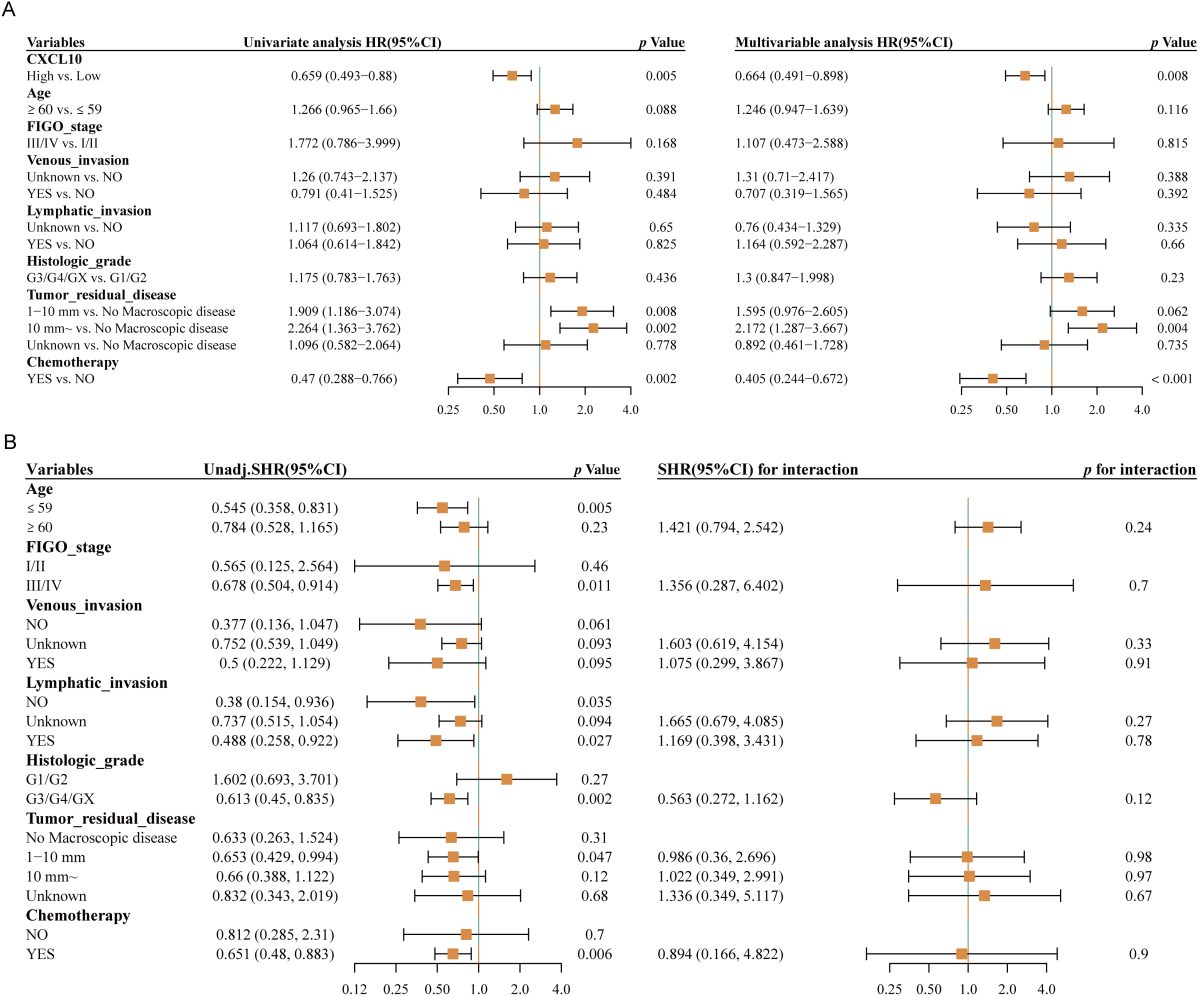
The analyses of univariate and multivariate Cox regression, exploratory subgroup, and interaction test. (A) Representative graph showing the results from the analyses of multivariate and univariate Cox regression. (B) Representative graph showing the analyses of exploratory subgroup and interaction test.

As shown in Figure 4A, chemotherapy (HR = 0.47, 95% CI: 0.288–0.766, *p* = 0.002) and high *CXCL10* expression (HR = 0.659, 95% CI: 0.493–0.88, *p* = 0.005) were protective factors for OS, while tumor residual disease was a risk factor (HR = 1.909, 95% CI: 1.186–3.074, *p* = 0.008; HR = 2.264, 95% CI: 1.363–3.762, *p* = 0.002) in univariate COX regression analysis. The multivariate analysis (Figure 4A) demonstrated that *CXCL10* expression can be used as an independent factor of prognosis for OC (HR = 0.664, 95% CI: 0.491–0.898, *p* = 0.008). Moreover, these subgroups (Figure 4B), such as age ≤ 59 years (HR = 0.545, 95% CI: 0.358–0.831, *p* = 0.005), FIGO stage III/IV (HR = 0.678, 95% CI: 0.504–0.914, *p* = 0.011), lymphatic invasion (HR = 0.488, 95% CI: 0.258–0.922, *p* = 0.027), histologic grade G3/G4/GX (HR = 0.613, 95% CI: 0.45–0.835, *p* = 0.002), and chemotherapy (HR = 0.651, 95% CI: 0.48–0.883, *p* = 0.006), high *CXCL10* expression can be used as a factor of protection for OS. As shown in Figure 4B, the association between *CXCL10* and OS in patients had no significant interactions with chemotherapy, histologic grade, lymphatic invasion, FIGO stage, or age (*p* > 0.05).

**FIGURE 5:**
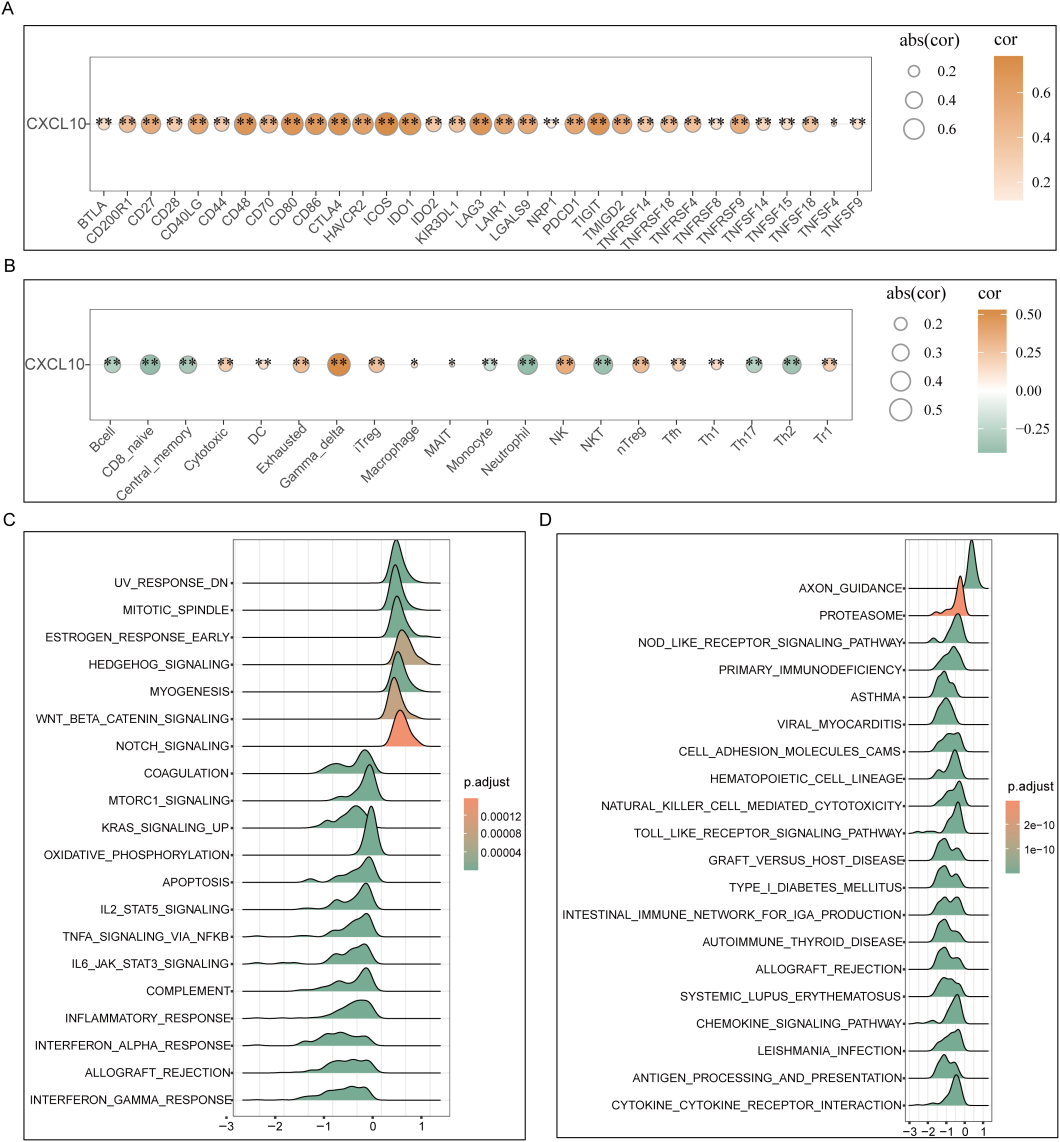
Immune checkpoints, immune cell infiltrations, and GSEA. (A) *CXCL10* expression positively correlated with immune checkpoints. (B) Differential immune cell infiltrations between the patients with low or high *CXCL10* expression. (C) GSEA based on Hallmark datasets. (D) KEGG analysis.

According to Spearman's correlation coefficient, a significantly positive correlation of *CXCL10* expression with immune checkpoints expression, such as CD80, CD86, and LAG3, was observed (Figure 5A *p* < 0.01). The number of NK, iTreg, cytotoxic, Tr1, and Tfh immune cells increased in the *CXCL10*‐high expression group. Furthermore, *CXCL10* expression was significantly positively correlated with the degree of immune cell infiltration (Figure 5B *p* < 0.01). Using the GSEA with the Hallmark and KEGG gene sets, significant enrichments of the genes differentially expression between the groups with low or high *CXCL10* expression high in Wnt/β‐catenin signaling, Notch signaling, Hedgehog signaling, and NOD‐like‐receptor signaling pathway were observed (Figure 5C,D).

### 3.2 Radiomics Feature Selection and Model Construction

**FIGURE 6:**
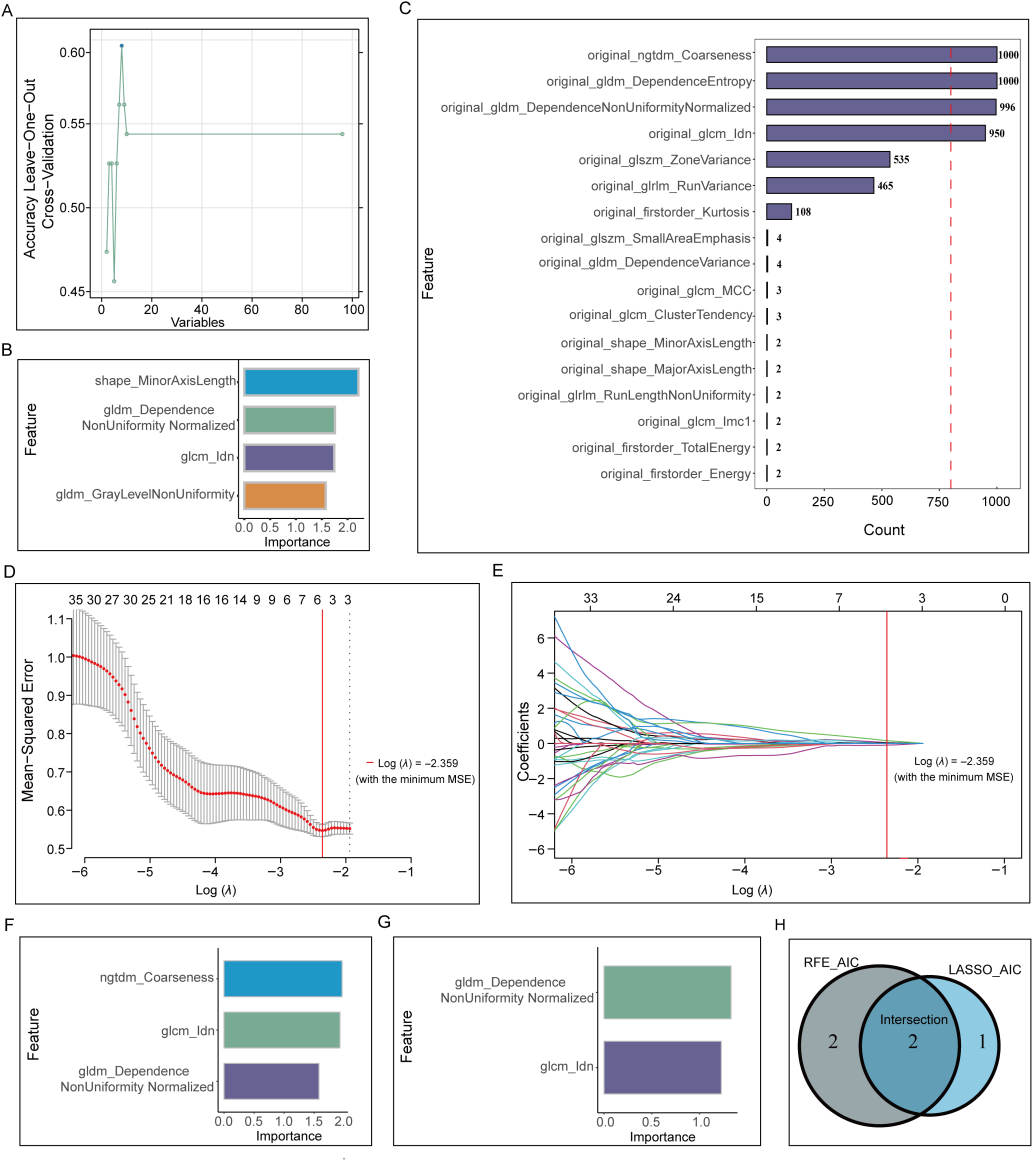
Radiomics feature screening and model building. (A, B) Representative graphs showing the RFE_AIC_LR radiomics signature established according to the RFE and logistic regression algorithms. (C–F) The LASSO_AIC_LR radiomics model was built using the LASSO and logistic regression algorithms. (G, H) The RFE_LASSO_LR model was constructed using the RFE and LASSO algorithms.

The 57 OC samples obtained from the intersection of TCGA and TCIA data were used as the research objectives. Ninety‐six radiomics features with ICC values ≥ 0.80 (median ICC of 0.965) were screened after data standardization. These features were used for subsequent screening, as shown in Table S1. For the evaluation of *CXCL10* expression, we employed the RFE and logistic regression algorithms to select four features and construct the RFE_ AIC_LR radiomics signature (Figure 6A,B). The three radiomic features selected via LASSO were fitted using a logistic regression algorithm to establish the LASSO_AIC_LR radiomics model for predicting *CXCL10* expression (Figure 6C–F). Two common radiomic features screened using the RFE and LASSO algorithms were used to construct the RFE_LASSO_LR model (Figure 6G,H).

### 3.3 Assessment of the Radiomics Model

**FIGURE 7:**
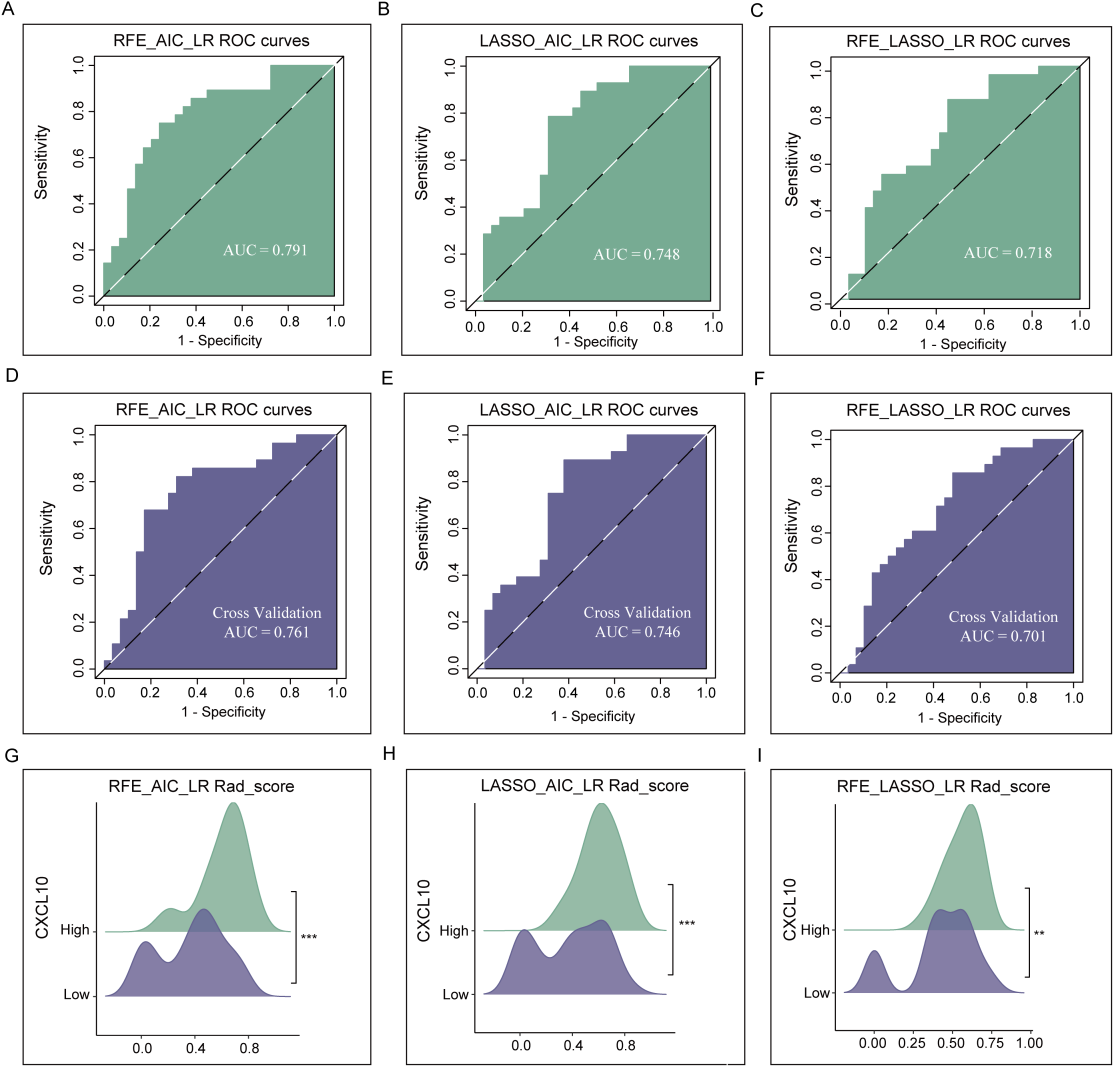
Assessment of the radiomic models to predict *CXCL10* expression. (A–C) Representative graphs showing the values of AUC and the curves of ROC in the training set. (D–F) Representative graphs showing the values of AUC and the curves of ROC in the validation set. (G–I) Rad‐score prediction of *CXCL10* expression level. Rad‐score, radiomics score.

The NPV, PPV, specificity, sensitivity, accuracy, and Brier scores of RFE_AIC_LR, LASSO_AIC_LR, and RFE_LASSO_LR are shown in Table 2. ROC curves showed AUC values for RFE_AIC_LR, LASSO_AIC_LR, and RFE_LASSO_LR in the training set of 0.791 (95% CI: 0.671–0.91), 0.748 (95% CI: 0.671–0.91), and 0.718 (95% CI: 0.583–0.853). In the set of validation, the values of AUC were 0.761 (95% CI: 0.631–0.891), 0.746 (95% CI: 0.616–0.877), and 0.701 (95% CI: 0.563–0.839) (Figure 7A–F).

**TABLE 2:** Evaluation of the radiomics signatures' efficacy in the sets for validation and training.

	RFE_AIC_LR		LASSO_AIC_LR		RFE_LASSO_LR	
	Training set	Validation set	Training set	Validation set	Training set	Validation set
Accuracy	0.754	0.754	0.737	0.754	0.702	0.684
Sensitivity	0.75	0.821	0.786	0.893	0.857	0.857
Specificity	0.759	0.69	0.69	0.621	0.552	0.517
PPV	0.75	0.719	0.71	0.694	0.649	0.632
NPV	0.759	0.8	0.769	0.857	0.8	0.789
Brier score	0.191	0.206	0.191	0.199	0.214	0.221

Abbreviations: NPV, negative predictive value; PPV, positive predictive value.

**FIGURE 8:**
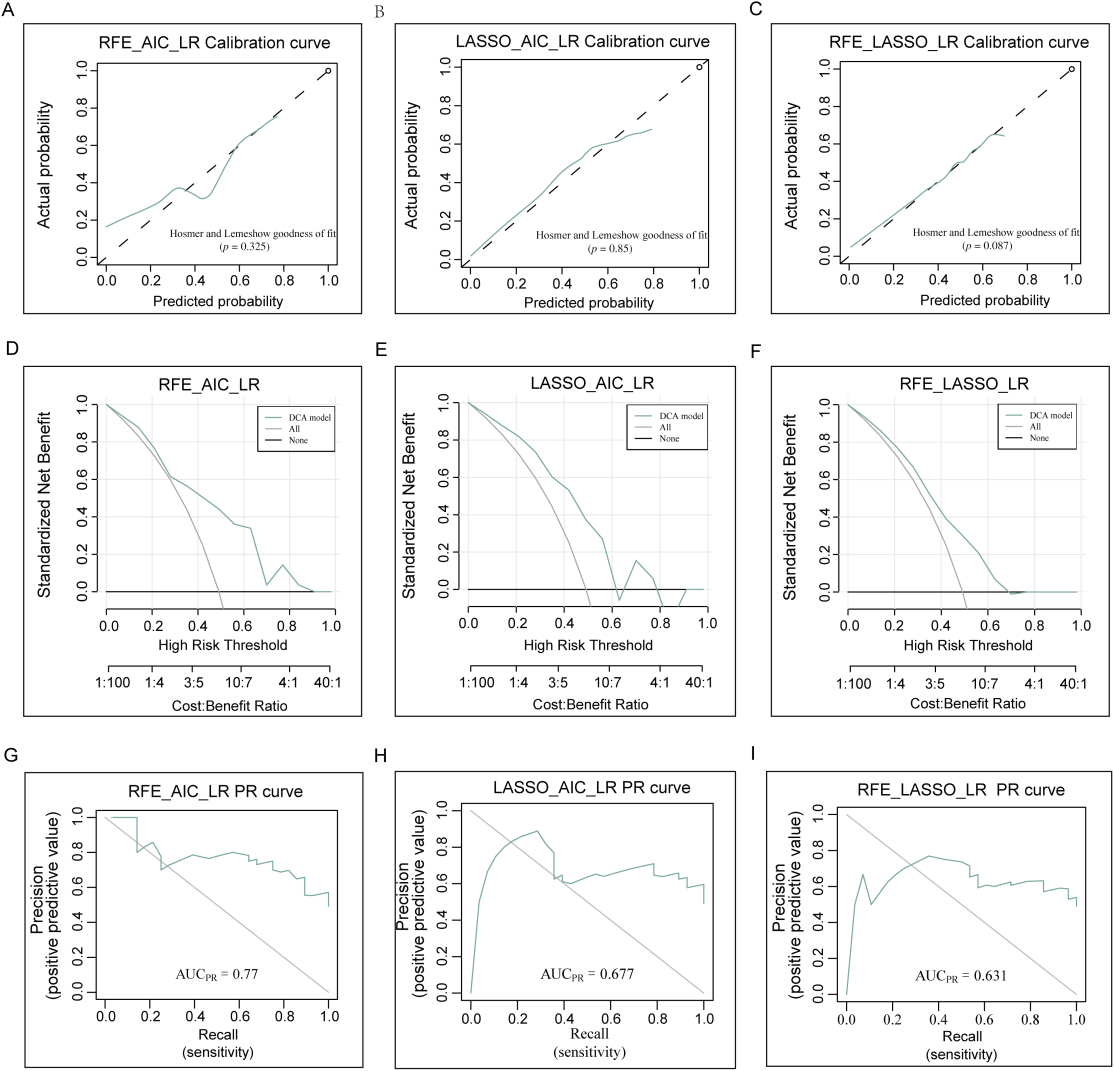
Assessment of radiomics signatures. (A–C) Representative graphs showing the curves of calibration. (D–F) Representative graphs showing the curves of DCA. (G–I) Representative graphs showing the curves of PR. Abbreviations: DCA, decision curve analysis; PR, precision‐recall.

Furthermore, the Hosmer–Lemeshow test and calibration curves indicated that the *CXCL10* expression predicted by the three radiomics models was consistent with the actual gene expression (*p* = 0.325; *p* = 0.85; *p* = 0.087, Figure 8A–C). As shown in Figure 8D–F, DCA graphically displayed the practical value of the radiomics models in a clinical setting. No significant difference in the values of AUC was observed after cross‐validation using the Delong test (*p* = 0.740, *p* = 0.989, and *p* = 0.863), indicating the good fit and predictive effect of the radiomics model. Radiomics prediction models observed a significant association of the Rad‐score with *CXCL10* expression by the Wilcoxon rank‐sum test (each *p* < 0.01, Figure 7G–I). The AUC values before and after the cross‐validation of each model showed their good prediction efficiency (Table 3). The PRs of the three models were 0.77, 0.677, and 0.631 (Figure 8G–I). Based on the AUC and PR values, the RFE_AIC_LR model was superior to the other two models after cross‐verification.

**TABLE 3:** The ability of radiomics models to make predictions was compared using AUC values.

	RFE vs LASSO	RFE vs RFE_LASSO	LASSO vs RFE_LASSO
Training set	0.333912	0.228036	0.601238
Validation set	0.746696	0.534798	0.639519

## 4 Discussion

Due to the heterogeneity, OC continues to pose a significant threat to human health, despite the considerable advances made in chemotherapy and surgery. Despite extensive progress in chemotherapy and surgery, OC remains one of the most lethal gynecological cancers worldwide owing to tumor heterogeneity. Immunotherapy is frequently applied in the treatment and therapy of OC [36]. However, the response of individual patients with OC varies, and the markers which could accurately predict the OS and response rate of OC to treatment are urgently needed [37].

*CXCL10* influences the microenvironment of intrahepatic tumor, particularly the immune response associated with tumor in the process of carcinogenesis. Tumor‐infiltrating cells (including NK, CD4^+^, and CD8^+^ cells), which negatively associated with the growth of tumor, were associated with increased *CXCL10* levels [38, 39, 40]. CXCL10 may provide new therapeutic options to improve cancer treatment by targeting the tumor microenvironment in multiple cancers [41]. The poor prognosis is significantly connected to the low *CXCL10* expression in CRC, and low *CXCL10* expression indicates adverse outcomes of CRC patients in stage II and III [42]. Li et al. [43] demonstrated that in rectal cancer, high CXCL10 expression is closely associated with better response to neoadjuvant chemoradiotherapy. In addition, significantly improved therapeutic efficacy was also observed in the murine xenograft tumor models after combined therapy of radiotherapy and CXCL10 gene therapy [44]. A positive correlation has been found between increased *CXCL10* expression and improved prognosis in various human cancers, including CRC, esophageal squamous cell carcinoma, hepatocellular carcinoma and breast cancer [16, 45, 46, 47]. Furthermore, elevated CXCL10 expression is positively correlated with a favorable prognosis in patients with OC [15, 48, 49]. Herein, significant overexpressed CXCL10 was observed in patients with OC at the transcriptional level. Additionally, we also found that CXCL10 expression can predict the prognosis of patients with OC independently (HR = 0.664, 95% CI: 0.491–0.898). Our study demonstrated a protective effect of *CXCL10* expression on the prognosis of patients with OC. Furthermore, *CXCL10* may play a crucial role in Wnt/β‐catenin, Notch, Hedgehog, and NOD‐like receptor signaling pathways, as shown in the enrichment analysis. Aberrant activation of these signaling pathways is associated with increasing prevalence, progressing malignancy, poorer prognoses, and even higher cancer‐related mortality rates [50, 51, 52, 53]. Accumulating evidence suggests that *CXCL10* can be an effective prognostic biomarker for OC, [15, 48, 49] which is worthy of in‐depth research.

Radiomics has been proposed as an emerging field for assessing the heterogeneity of tumor burden in OC [28]. Nougaret et al. [54] reported that qualitative CT feature analysis of patients with *BRCA* mutations and wild‐type high‐grade serous OC revealed different characteristics and could predict OS. Rizzo et al. [55] analyzed CT radiomic features in 101 patients with OC and found radiological features related to homogeneity, randomness, and mass size, which were associated with the remaining tumor characteristics. A recent study showed that the radiomic signature of CT images could be used to determine *PD‐1* expression status and the prognosis of patients with OC [56]. Using this model, the values of AUC for the training and validation sets were obtained as 0.810 (95% CI: 0.696–0.924) and 0.771 (95% CI: 0.645–0.899). Herein, we established and validated radiomics models based on enhanced CT radiomics to predict *CXCL10* expression levels. In addition, three radiomics models were built using RFE, LASSO, and logistic regression algorithms, whose values of AUC in the training set were 0.791, 0.748, 0.718, as well as 0.761, 0.746, and 0.701 in the validation set, respectively. Meanwhile, high Rad‐scores were associated with elevated *CXCL10* expression. Although radiomics models are not widely used in clinical work at present, there is great expectation for their future clinical applications. With the continuous progress of technology and in‐depth research, radiomics models are expected to play a crucial role in clinical diagnosis, treatment decision‐making, and prognosis assessment. However, to achieve the wide application of radiomics models in clinical practice, some challenges still need to be overcome. For instance, it is necessary to further enhance the accuracy and reliability of the model to ensure its stability in different medical institutions and among patient groups. At the same time, unified standards and specifications need to be established to facilitate the sharing and exchange of radiomics data and promote the rapid development of this field.

However, limitations are also existed. First, the data used in this study are from different research institutions, and the differences in imaging parameters between institutions inevitably lead to significant variations in the data and images. In future research, we will incorporate more imaging data from our medical center or multiple institutions. Through setting reasonable acquisition parameters and establishing a unified quality control standard, we can calibrate and normalize the data collected by different scanners, thus reducing the disparities in data and images. Second, owing to the strict criteria of exclusion/inclusion, only 57 images were obtained, and the sample size was small. The radiomic features still require further verification using large‐sample and multicenter research. Third, in this study, we only employed internal cross‐validation and did not perform external validation with an independent dataset. In subsequent studies, we will actively seek appropriate independent datasets for external validation, including data of patients with OC from our research center or public databases such as ICGC and SEER, to ensure the accuracy and reliability of the model. Meanwhile, we will regularly collect updated data of patients with OC and retrain and optimize the model. Researchers have found that when CT‐based radiomics features are combined with traditional clinical parameters, it can enhance the predictive value of prognosis for the survival of patients with different malignant tumors [57]. Finally, we will consider combining the radiomic features extracted from CT images with pathology and serum markers, such as *CA125* and *HE4*, to establish a more comprehensive radiomics model in the future.

## 5 Conclusions

In summary, the radiomic models presented here can evaluate the *CXCL10* expression status via CT imaging, which can indirectly reflect the prognosis of patients with OC. Rad_score is an effective prediction tool, which can assist clinical decision‐making and guide individualized precision diagnosis and treatment. Radiomic signatures may provide a noninvasive and reliable supplementary method for the prognostic prediction of the patient with OC in clinics. With the advent of the era of big data, radiomics development, and precision medicine demand, the combined application of proteomics, genomics, and radiomics may be a new direction for future research.

## Author Contributions

**Xiaohua Wang:** conceptualization, methodology, investigation, formal analysis, funding acquisition, writing – original draft, writing – review and editing, data curation, visualization, project administration. **Yuanyuan Xing:** conceptualization, validation, investigation, visualization. **Xuan Zhou:** methodology, software, validation, resources. **Chunhui Wang:** methodology, validation, investigation, resources, data curation. **Shuyu Han:** software, validation, resources, visualization. **Sufen Zhao:** conceptualization, writing – review and editing, supervision, project administration, formal analysis.

## Ethics Statement

All data and images from TCGA and TCIA were anonymized, and thus, no written informed consent and review from ethical committee are needed.

## Conflicts of Interest

The authors declare no conflicts of interest.

## Supporting information


Data S1.

## Data Availability

The data that support the findings of this study are available from the corresponding author upon reasonable request.
