# Supplementary material for: Radiomics Signatures Based on Computed Tomography for Noninvasive Prediction of CXCL10 Expression and Prognosis in Ovarian Cancer
Source: Cancer Rep (Hoboken). 2024 Oct 23;7(10):e70030. doi: 10.1002/cnr2.70030 (PMC11499071; doi:10.1002/cnr2.70030)
Supplement: Supplementary file 1 — Data S1. [file CNR2-7-e70030-s001.docx]

**Table S1.** Consistency evaluation of intraclass correlation efficient.

|  | **ICC ≥ 0.8** | **0.5 ≤ ICC < 0.8** | **ICC < 0.5** | **ICC_Mean** | **ICC_Median** |
| --- | --- | --- | --- | --- | --- |
| Percentage | 0.897 | 0.075 | 0.028 | 0.922 | 0.965 |
| Number | 96 | 8 | 3 | NA | NA |
